# Supplementary material for: Diagnostic, prognostic, and therapeutic potentials of gut microbiome profiling in human schistosomiasis: A comprehensive systematic review
Source: PLoS Negl Trop Dis. 2025 Feb 3;19(2):e0012844. doi: 10.1371/journal.pntd.0012844 (PMC11844881; doi:10.1371/journal.pntd.0012844)
Supplement: S2 Table — (DOCX) [file pntd.0012844.s002.docx]

**S2 Table: Search strategy through Medline, Embase, Global Health, Web of Science, and Global Index Medicus databases**

| **Database** |  | Search | Items |
| --- | --- | --- | --- |
| **Medline (Ovid)** | 1 | exp Gastrointestinal Microbiome/ | 36945 |
|  | 2 | (Gut microbiome or Gastrointestinal Microbiome or Gastrointestinal Microbiomes or Gut microbiomes or Gut microflora or Gut Microbiota or Gastrointestinal Flora or Gut Flora or Gastrointestinal microbiota or Gastrointestinal Microbial Community or Gastrointestinal Microflora or Gastric microbiome or Gastric microbiomes or Intestinal Microbiome or Intestinal Microbiota or Intestinal Microflora or Intestinal Flora or Enteric bacteria).mp. | 63386 |
|  | 3 | exp Schistosomiasis/ or exp Schistosomiasis haematobia/ or exp Schistosomiasis mansoni/ or exp Schistosomiasis japonica/ or exp Bulinus/ or exp Biomphalaria/ or exp Hypertension, Portal/ | 54526 |
|  | 4 | (Schistosomiasis or Schistosomiases or Schistosomiasis haematobia or Schistosomiasis mansoni or Schistosomiasis japonica or Schistoma Infection or Katayama Fever or Bilharzia or Bilharziasis or Bilharziases or Bulinus or Biomphalaria or Portal hypertension).mp. | 48216 |
|  | 5 | 1 or 2 | 63386 |
|  | 6 | 3 or 4 | 62394 |
|  | 7 | 5 and 6 | 119 |
|  |  |  |  |
| **Embase (Ovid)** | 1 | exp intestine flora/ | 96156 |
|  | 2 | (intestine flora or gut microbiome or gastrointestinal microbiome or gastrointestinal microbiomes or gut microbiomes or gut microflora or gut microbiota or gastrointestinal flora or gut flora or gastrointestinal microbiota or gastrointestinal microbial community or gastrointestinal microflora or gastric microbiome or gastric microbiomes or intestinal microbiome or intestinal microbiota or intestinal microflora or intestinal flora or enteric bacteria).mp. | 121917 |
|  | 3 | exp schistosomiasis/ or exp schistosoma/ or exp intestinal schistosomiasis/ or exp schistosomiasis haematobia/ or exp schistosomiasis mansoni/ or exp schistosomiasis japonica/ or exp bulinus/ or exp biomphalaria/ or exp portal hypertension/ | 73497 |
|  | 4 | (schistosomiasis or schistosomiases or schistosoma or schistosomiasis haematobia or schistosomiasis mansoni or schistosomiasis japonica or schistoma infection or katayama fever or bilharzia or bilharziasis or bilharziases or bulinus or biomphalaria or portal hypertension).mp. | 80844 |
|  | 5 | 1 or 2 | 123630 |
|  | 6 | 3 or 4 | 83022 |
|  | 7 | 5 and 6 | 415 |
|  |  |  |  |
| **Global Health (Ovid)** | 1 | (intestine flora or gut microbiome or gastrointestinal microbiome or gastrointestinal microbiomes or gut microbiomes or gut microflora or gut microbiota or gastrointestinal flora or gut flora or gastrointestinal microbiota or gastrointestinal microbial community or gastrointestinal microflora or gastric microbiome or gastric microbiomes or intestinal microbiome or intestinal microbiota or intestinal microflora or intestinal flora or enteric bacteria).mp. | 36632 |
|  | 2 | (schistosomiasis or schistosomiases or schistosoma or schistosomiasis haematobia or schistosomiasis mansoni or schistosomiasis japonica or schistoma infection or katayama fever or bilharzia or bilharziasis or bilharziases or bulinus or biomphalaria or portal hypertension).mp. | 56696 |
|  | 3 | 1 and 2 | 80 |
|  |  |  |  |
|  |  |  |  |
| **Web of Science** | 1 | Topic = (intestine flora or gut microbiome or gastrointestinal microbiome or gastrointestinal microbiomes or gut microbiomes or gut microflora or gut microbiota or gastrointestinal flora or gut flora or gastrointestinal microbiota or gastrointestinal microbial community or gastrointestinal microflora or gastric microbiome or gastric microbiomes or intestinal microbiome or intestinal microbiota or intestinal microflora or intestinal flora or enteric bacteria) AND (schistosomiasis or schistosomiases or schistosoma or schistosomiasis haematobia or schistosomiasis mansoni or schistosomiasis japonica or schistoma infection or katayama fever or bilharzia or bilharziasis or bilharziases or bulinus or biomphalaria or portal hypertension) | 261 |
| **Global Index Medicus** |  | (intestine flora or gut microbiome or gastrointestinal microbiome or gastrointestinal microbiomes or gut microbiomes or gut microflora or gut microbiota or gastrointestinal flora or gut flora or gastrointestinal microbiota or gastrointestinal microbial community or gastrointestinal microflora or gastric microbiome or gastric microbiomes or intestinal microbiome or intestinal microbiota or intestinal microflora or intestinal flora or enteric bacteria) AND (schistosomiasis or schistosomiases or schistosoma or schistosomiasis haematobia or schistosomiasis mansoni or schistosomiasis japonica or schistoma infection or katayama fever or bilharzia or bilharziasis or bilharziases or bulinus or biomphalaria or portal hypertension) | 6 |
