# Supplementary material for: Diagnostic, prognostic, and therapeutic potentials of gut microbiome profiling in human schistosomiasis: A comprehensive systematic review
Source: PLoS Negl Trop Dis. 2025 Feb 3;19(2):e0012844. doi: 10.1371/journal.pntd.0012844 (PMC11844881; doi:10.1371/journal.pntd.0012844)
Supplement: Attachment — Submitted filename: Answers_to_reviewers.docx [file pntd.0012844.s004.docx]

**Answers to Reviewers**

Methods

Reviewer #3: There are several issues with the Methods section:

1. In the "Assessment of Risk of Bias," the authors should specify which JBI checklists were used. Since they included various types of study designs, these cannot be assessed with a single quality assessment checklist. Additionally, because they incorporated websites into their search, they should clarify the details of the findings derived from these websites, as well as the checklist used to assess their quality.

**Authors: We appreciate the reviewer’s concern. We have updated the information to indicate that we used an assessment tool for cross-sectional prevalence data adapted from the tool developed by Hoy and colleagues, which evaluates both the external and internal validity of the included studies. Regarding the search of studies on websites, it was actually a citation search, which we have now corrected in both Figure 1 and the study selection description.**

**The critical appraisal tool developed by Hoy and colleagues was used to assess the quality of the studies. This tool, specifically designed for cross-sectional prevalence studies, evaluates both external and internal validity. If the study was not excluded, a score was calculated for each article and classified as “high,” “moderate,” or “low” risk of bias (S3 Table).**

2. The PRISMA flowchart (Figure 1) requires further clarification regarding "title and abstract screening," "full text screening," and "included studies." It is unclear why the flowchart mentions both "studies included in review (n=13)" and "reports of included studies (n=13)" in the final section.

**Authors: We appreciate the reviewer’s concern. We updated the description of the study selection. We also updated Figure 1 as shown below.**

**As shown in Figure 1, the literature search across various databases initially yielded a total of 881 potentially relevant articles. After removing 302 duplicate articles and excluding 555 articles based on a thorough screening of titles and abstracts, 24 articles were sought for retrieval. Of these, 3 articles could not be retrieved, leaving 21 articles to be assessed for eligibility. Following the full-text screening, 9 articles met the inclusion criteria. Additionally, 4 relevant articles were identified through manual searches of the reference lists of the included studies, resulting in a total of 13 publications included in this systematic review.**

--------------------

Results

Reviewer #2: Most sections of the results section must be rewritten

**Authors:**

**We appreciate the reviewer’s concern. We performed revisions of the result section as much as possible and had it re-read by several departmental colleagues to ensure fluency to a large number of readers from different backgrounds. We do hope the present version of the manuscript to be satisfactory to this reviewer. In fact, we also do build on specific requests from reviewer 3 below on this result section to efficiently guide the modifications.**

Reviewer #3: There are several issues with the Results section:

1. There are missing references throughout the Results section. The authors should cite the references for the included studies when reporting their characteristics. For example, in line 235, they should reference the "cross-sectional studies" when mentioning their number.

2. In line 242, the sentence "In SSA, the most affected part worldwide" is not a result of their study. The authors should either provide a reference for this statement or delete it, as the Results section should only include findings from the included studies.

3. There is a significant amount of heterogeneous information in the tables. For example, in Table 1, it is expected to summarize the "study population" coherently (e.g., how many patients were examined). Additionally, there is missing information regarding the demographic characteristics of the studied population (e.g., gender, age, etc.) that should be presented in separate columns in Table 1. Table 2 also contains excessive uncategorized information. In systematic reviews, it is important to present principal information clearly and concisely in tables.

4. Figure 2 has very low quality and should be enhanced for clarity.

5. There are several misclassifications of bacterial taxa. For example, in line 358, "Neisseriales," "Rickettsiales," and "Fusobacteriales" are not classes but orders. The authors should review the manuscript carefully and address all necessary corrections.

6. Correct notation should be used for bacterial taxa. For instance, in line 392, "Firmicutes" is a kingdom and should not be italicized. There are many other items to address. The authors can refer to "https://wwwnc.cdc.gov/eid/page/scientific-nomenclature" for guidance on reporting bacterial names. Note that kingdom, phylum, class, order, and suborder should begin with a capital letter but should not be italicized.

**Authors:**

1. **We agree with the reviewer. In the revised version, references were included throughout the Results section mainly in lines 236, 237, 238, 239, 241, 243, 244, 247, 248, 249, 251, 252, 257, and 258.**
2. **In the sentence “**In SSA, the most affected part worldwide**…”, we tried to emphasize the fact that, SSA where the burden of Human schistosomiasis is greatest and endemic in forthy countries, studies were carried out only in 3 countries. In the revised version, we rephrased the sentence and added a reference. See lines 244-249.**
3. **In table 1, we summarized the characteristics of all studies included in this systematic review. Columns 6 from the left contains description of the studies population. Data included are reported according of the information given by authors of every included study. Nevertheless, we added, as requested by this reviewer, any missing information regarding the sociodemographic characteristics of studies populations when it was possible to access those data in the article (see line 264). In table 2, we now modified to show concisely only the main genera that are altered in Schistosoma-infected subjects. See line 386.**
4. **We agree with the reviewer and have enhanced the clarity of the figure 2 in-text and also provided an additional pdf version of the figure 2, to be used to generate a high-resolution tiff file, should the paper be accepted.**
5. **Bacteria classification was corrected in line 363, we agree to the mistake from our end and did replace class with order as rightfully pointed out by this reviewer. See line 363**
6. **We also thank the reviewer for the input here and provided link. Adequate corrections were made in that respect in lines: 339, 340, 342, 344, 346, 363, 397.**

--------------------

Conclusions

Reviewer #2: The conclusion cannot be matched with the objective of the study as well as with the title of the manuscript

**Authors:**

**The Objective of the review outlined in lines 133-135 on elucidating the current relationship between the gut microbiome and schistosomiasis in humans and thereby assess the diagnostic, prognostic and therapeutic potential of any eventual particularity of the human host gut microbiome during schistosomiasis (reported as the title here on the premise of this overall objective) has been tentatively answered in the conclusion in a cautious manner without already giving here a definitive list of microbes with diagnostic, prognostic or therapeutic values from the human gut microbiome during schistosomiasis. Such an approach would have prematurely raised observations from accumulating studies as the general profile until such time large and longitudinal validation are made.**

**Our systematic review, though presenting some of these elements from individual studies run on high throughput screening techniques (still needing validation from more specific techniques and settings i.e. longitudinal versus transversal etc..), ultimately, but cautiously, answer the question in the conclusion lines 487 – 492 by unambiguously stating that human schistosomiasis indeed alters in a singular manner the gut microbiome and that the nascent observation of shared alterations between infected individuals suggest indeed a potential to use the gut microbiome of schistosomiasis diseased individuals to determine diagnostic prognosis and/or therapeutic biomarkers of the disease. Delving deeper than this is not yet unequivocally substantiated by the current literature, though we do provide in simple terms a clear answer to the title and objective that indeed diagnostic prognosis and therapy of human schistosomiasis can be biomark by the gut microbiome and that some preliminary evidences of that are now been consolidated in independent studies.**

**As such, we do hope this reviewer to approve our cautious approach in answering the question, raised by the present objective and study title and the clear flow of logic between objective, title and conclusion herein presented in our review.**

Reviewer #3: There are several issues with the Discussion and Conclusions sections:

1. The Conclusion section is too lengthy. I suggest summarizing this part to highlight the key findings of the study.

2. In lines 387-389: "In recent decades, several studies were undertaken to characterize the gut microbiome signature of schistosomiasis-infected patients with or without associated liver pathology." Please provide references for this statement and cite the mentioned studies.

**Authors:**

1. **The conclusion section was separated from the outlook and cut down from 262 words to 73 words stating concisely the findings from our systematic review. See lines 487-492. A new section on outlook of the study, stating the limitations and way forward was created. See line2 496-514.**
2. **Citation in line is now available in the revised manuscript, in line 393.**

--------------------

Editorial and Data Presentation Modifications

Use this section for editorial suggestions as well as relatively minor modifications of existing data that would enhance clarity. If the only modifications needed are minor and/or editorial, you may wish to recommend “Minor Revision” or “Accept”.

Reviewer #1: Line 41: replace "i.e" with "that is"

Line 69: This neglected tropical disease affects atleast 251.4 million people

Line 230: Replace 'On' with 'Of' in the sentence "Of the 24 articles...."

Line 390: Also add that "in order to determine their diagnostic, prognotsic and therapuetic potential."

**Authors:**

- **“i.e” was replaced with “that is” in line 41**
- **“effected” was replaced with “affects” in line 69**
- **We rephrase the sentence “Of the 24 article…”**
- **In line 391, we have replaced “because it is considered as source of potential biomarkers for diseases diagnostics, monitoring and development of new medical treatment” with “in order to determine their diagnostic, prognostic and therapeutic potential."**

Reviewer #2: I can submit the PDF of this manuscript in which the minor and majors points have been underlined

**Authors:**

**All points raised by this reviewer, though generic for some, have been considered to comprehensively revise the submission and address, in accordance with all other reviewers’ comments, the possible limitations of the initial submission.**

Reviewer #3: The manuscript would benefit from language revisions to enhance fluency. For example, in line 385, the phrase 'The studying of human gut microbiome has received increasing attention because...' is awkward. It can be modified to 'Human gut microbiome studies have garnered increasing attention in recent years.' Please review and revise the manuscript accordingly.

**Authors: We carefully revised the manuscript to enhance the fluency. Several typos and poorly structured sentences have tried to be addressed eg. Lines 41, 69, 133-135, 185-188, 228-234, 244-245, 390-391.**

Summary and General Comments

Reviewer #1: The systematic review was well written however a few points that need to be included in the conclusion;

- A need to discuss the data that was missing from these studies which would influence the outcome. For instance, the general assumption is that the participants/patients had monoinfection and hence no report on the cofounder effects of comorbidities and confections that could potentially influence the outcome.

- The diet of the participants. The diet plays a key role in the structure of the gut microbiome. The food composition was worth reporting since this is not a constant for all the communities from the different geographical locations

**Authors**

**This is indeed a valid point and the reason for us not to prematurely report the observed changes in the included studies as general consensus changes resulting solely from schistosomiasis. We have made mention of the need to expand the datasets around the study of the impact of the gut microbiome during human schistosomiasis to more clinical settings including diet-controlled and poly- versus mono-infection settings. See line 500-501 of the outlook.**

Reviewer #2: This manuscript PNTD-D-24-01193 of Oyono et al entitled "Diagnostic, Prognostic, and Therapeutic Potentials of Gut Microbiome Profiling in Human Schistosomiasis: A Comprehensive Systematic Review" treat an interesting topic by reviewing published article dealing with microbiome modifications during schistosome infections.

Although the title is very interesting, the manuscript content does not reflect the title. In the manuscript, the authors tried to identify the diversity of microorganims between schistosomiasis infected and uninfected and also between patients presenting different schistosomiasis-related pathologies. These authors also tried discuss the impacts of the presence of some specific microorganisms with immnunological and clinical outcome of schistosome infections in patients. The pathological implications of the presence of these microorganisms were also discussed. In terms of diagnostic, prognostic and therapeutic potentials of these microorganisms, no data can be inferred. I was expected to see a list of potential biomarkers that could open a framework for investigations aiming to develop new diagnostic or prognostic or therapeutic tools. In their study, the authors reported diversity of gut microorganisms with some of them that are probably abundant when the infected patients

present some pathological manifestations. However, as these microorganisms could be found in patients suffering from other diseases, could their detection be considered as potential indication of schistosome infections and the pathology associated to these infections? This article could be focused on : A Comprehensive Systematic Review on gut Microbiome Profiling in Human Schistosomiasis for the understanding of schistosome infections and associated pathologies.

The manuscript is not well written and the language requires deep revision

Overall, this manuscript contains a lot of shortcomings and in the present form, it cannot be accepted for publication in Plos NTD

**Authors: All points relating to the apparent lack of fluency have been reconsidered in depth and conclusions rendered more succinct to clearly align with the study title and objective, while remaining cautious enough not to erroneously report the observed changes, though independently confirmed for some geographically and culturally distant studies, as consensus for human patients infected by schistosomiasis (see specific answers to this reviewers’ queries).**

**Our revised manuscript now cautiously and robustly report that changes occur as a result of schistosomiasis infection, infection progression and response to treatment suggesting broadly a role of the gut microbiome in biomarking diagnosis, prognosis and therapy during human schistosomiasis. Beyond such an apparent potential, our work which comprehensively reported all such changes, refrain from pinpointing the microbial biomarkers as definitive but strongly advocate for the expansion of the available data sets of studies, focusing on large, longitudinal, diet-controlled and poly-infected clinical settings on well phenotyped participants to robustly feed an accepted list of true microbial biomarkers.**

**Whatever the limitations, our systematic review remains unprecedentedly comprehensive and does provide a definitive answer in support of characterizing further the gut microbiome to help the diagnostic, prognosis and therapeutic ventures during human schistosomiasis.**

Reviewer #3: (No Response)
