# Supplementary material for: Diagnostic, prognostic, and therapeutic potentials of gut microbiome profiling in human schistosomiasis: A comprehensive systematic review
Source: PLoS Negl Trop Dis. 2025 Feb 3;19(2):e0012844. doi: 10.1371/journal.pntd.0012844 (PMC11844881; doi:10.1371/journal.pntd.0012844)
Supplement: Attachment — Submitted filename: Answers to Reviewers.docx [file pntd.0012844.s005.docx]

RESPONSE TO REVIEWERS

1. **Reviewers' comments**

**Editorial and Data Presentation Modifications**

**Reviewer #2:**

- Lines 115- 117: References must be added
- The size of figure must be increased
- Line 238-242: These sentences must be reviewed
- Line 244: One "burden" must be deleted
- Lines 345-347: This sentence must be reviewed

**Authors:**

We appreciate the reviewers’ corrections and have carefully revised our manuscript to address all of them effectively.

1. In line 117, we have added new references, and we’ve updated the remaining references throughout the manuscript.
2. The figure size has been enhanced in-text for better visibility and a high resolution version of the figures 1 and 2 provided for consideration and production processings
3. We have refined the sentences in lines 237-240 for improved clarity.
4. Additionally, we removed one occurrence of the word "burden" in line 243.
5. Finally, the sentence in lines 344-345 has been thoughtfully revised to enhance its impact.
